# Improving Postoperative Pediatric Recovery by Efficient Recovery Room Care—A Comprehensive Review

**DOI:** 10.3390/children12050568

**Published:** 2025-04-28

**Authors:** Lisa Korell, Frank Fideler

**Affiliations:** Department of Anesthesiology and Intensive Care Medicine, University Hospital, Eberhard Karls University Tuebingen, 72076 Tuebingen, Germany; frank.fideler@med.uni-tuebingen.de

**Keywords:** pediatric anesthesia, pediatric recovery room care, postoperative complications, post-anesthetic pain management, optimization options, organizational factors, interdisciplinary evidence implementation

## Abstract

**Background/Objectives**: Efficient postoperative recovery room care in pediatric patients is crucial for optimizing perioperative safety, patient outcome, and effective pain management. However, this area is frequently underemphasized, leading to higher complication rates compared to the operating room, which in turn increases healthcare costs. Improving pediatric recovery room care offers a significant opportunity to enhance the quality and safety of perioperative pediatric care. From an economic perspective, this is prudent; however, more importantly, every child has the right to the highest attainable standard of health, as outlined by the United Nations. Key aspects of recovery room care include ensuring adequate staffing and equipment, while also prioritizing the child’s privacy and parental presence, both of which are crucial for enhancing patient well-being. A +multimodal approach to postoperative pain management is essential for minimizing fear and stress, alongside strict adherence to established guidelines for the management of postoperative nausea, vomiting, and emergence delirium. Furthermore, addressing risk factors such as hypothermia and airway complications, as well as promoting early intake of clear fluids, plays a crucial role in optimizing pediatric recovery. Organizational strategies such as quality improvement initiatives, structured handovers, standardized care protocols with checklists, continuous staff training, and well-defined discharge criteria are further essential components to reduce translational gaps and to enhance postoperative pediatric safety. **Conclusions**: Improving pediatric postoperative anesthetic care is a multifaceted challenge for all healthcare providers that can significantly enhance care quality and safety while also reducing costs. Success in this area requires addressing both structural and medical factors.

## 1. Introduction

Postoperative anesthetic care for pediatric patients requires a highly specialized approach, tailored to their distinct physiological and psychological characteristics. Despite its critical importance, immediate postoperative care for children frequently suffers from insufficient attention by healthcare providers, with poorly defined treatment protocols and responsibilities often compounding the issue. Alarmingly, research by Murat et al. has highlighted a significantly higher complication rate in pediatric patients in recovery rooms (4.3%) compared to operating rooms (3.1%) [1]. As in adults, it must, therefore, be assumed that postoperative complications are common and result in increased healthcare costs [2,3], although the full scope of this challenge is often under-recognized [4].

The principles enshrined in the United Nations Convention on the Rights of the Child affirm that every child has the right to the highest attainable standard of health. This includes access to comprehensive pain management strategies aimed at alleviating pain, fear, and unnecessary stress during recovery. Furthermore, pediatric patients have the right to the presence of their parents during postoperative recovery and the assurance of privacy throughout their care [5].

Given the complexity and multifaceted nature of postoperative anesthetic care for children, there is a substantial opportunity to enhance both the safety and quality of pediatric care while simultaneously achieving better economic outcomes. Achieving these goals requires a wide range of structural, clinical, and psychosocial factors. This article aims to provide an in-depth exploration of the critical considerations necessary for optimizing safety and outcomes in pediatric patients within recovery rooms, offering a framework to advance the standard of care in this vital area of medicine, particularly for non-anesthesia medical providers.

## 2. Materials and Methods

The objective of this literature review was to summarize current treatment strategies for children in the recovery room, identify gaps in existing knowledge, and explore opportunities to improve both the safety and quality of pediatric post-anesthesia care.

We followed the IMRAD structure and conducted a systematic search of PubMed, Embase, Scopus, Web of Science, CENTRAL, and EudraCT/CTIS for published studies, and PROSPERO for completed systematic reviews, up to 17 April 2025.

Search terms included recovery room, postoperative complications, pain, anxiety, emergence delirium, and postoperative nausea and vomiting.

Inclusion criteria comprised studies published in English or German, focusing on pediatric populations, and including clinical trials, preclinical studies, observational studies, or meta-analyses. Preference was given to recent double-blind or single-center studies from Europe or North America with a low risk of bias. Additional references were identified using snowballing techniques.

Studies that were not published in full or were available only in other languages were excluded.

Both authors independently conducted the search and assessed the relevance of included studies. Pediatric and selected adult anesthesia studies were included following discussion. The extracted data were categorized thematically, as outlined in the subheadings of the Section 3, and are discussed therein.

## 3. Results

### 3.1. Post-Anesthetic Recovery Room

Providing postoperative care for pediatric patients requires a dedicated recovery unit equipped to address the unique challenges of this population [6]. By the critical incident technique, it could be shown that evidence-based care and safety are compromised when technology has limitations and is not adapted for pediatric use [7]. The specific resources and capabilities of the recovery unit should be adapted to the severity of the treated conditions and include the following fundamental components:

#### 3.1.1. Essential Infrastructure and Equipment

- Reliable access to oxygen, air, and vacuum systems.
- Tools and expertise for emergency airway management.
- Emergency medications and appropriately scaled dosing tools for pediatric use.
- Immediate access to diagnostic tools, including ultrasound and, where indicated, X-ray facilities.
- Facilities for blood gas analysis and access to clinical laboratories, with a blood bank available depending on the surgical procedures performed.

#### 3.1.2. Trained Personnel and Operational Standards

- Staff thoroughly trained in pediatric care, trained in recognizing the age-specific needs of children [8], and adhering to established standards while utilizing structured care protocols and checklists.
- A clear communication hierarchy with accessible contact information for all relevant personnel [9].
- Implementation strategies for tailoring existing evidence to the target population and effectively applying it in the local clinical setting [10,11,12].

#### 3.1.3. Comprehensive Patient Monitoring

- Pulse oximetry: Mandatory for all patients, with both pre- and post-ductal measurements recommended for infants younger than four weeks.
- Carbon dioxide monitoring: This should be performed via nasal cannula or transcutaneously when an impaired neurological status is present, in order to accurately detect hypoventilation or apnea before desaturation occurs [13].
- Electrocardiographic (ECG) monitoring: Performed based on clinical risk factors. For heart rates exceeding 200 beats per minute, a 12-lead ECG is essential to ensure accurate rhythm differentiation.
- Blood pressure: Measured on admission, before transfer, and at regular intervals based on the patient’s clinical status.
- Temperature: Monitored at a minimum upon admission and before transfer.
- Blood glucose: Regularly assessed in patients at risk for hypo- or hyperglycemia to maintain euglycemia [14] (Table 1).

**Table 1:** Risk factors for postoperative hypoglycemia and hyperglycemia in which meticulous monitoring in the recovery room is necessary [15,16,17,18,19].

Postoperative Risk for	Risk Factors
**Hypoglycemia** **in need of therapy for blood glucose values <60 mg/dL (<70 mg/dL in type 1 diabetes)**	○ Age < 12 months/weight for age <5th percentile developmental delay/failure to thrive ○ ASA status ≥ III ○ Long fasting times ○ Abdominal surgery ○ Metabolic disease ○ Low glycogen reserve (parenteral nutrition, liver disease, beta blockers, diabetic mother)
**Hyperglycemia/ketoacidosis** **in need of therapy for blood glucose values > 220–250 mg/dL**	○ Type I diabetes

##### Primary Objectives of Pediatric Recovery Care

The principal goals in the pediatric recovery room include the following:

1. Stabilizing vital parameters.
2. Providing effective pain management.
3. Timely detection and treatment of complications after anesthesia.
4. Minimizing emotional distress.
5. Reducing and treating nausea and vomiting.
6. Achieving an adequate level of consciousness.
7. Ensuring that the level of required monitoring and support is compatible with the capabilities of the ward where further care will be provided.

##### Environmental and Psychosocial Considerations

A calm, dimly lit environment is essential for reducing stress and anxiety, particularly in premature infants and neonates. Minimizing the frequency and invasiveness of interventions further supports a stress-free recovery process. The presence of parents in the recovery room is strongly recommended, as their observations provide valuable insights into the child’s condition, aiding recovery room staff in assessing the patient’s status [5].

A study surveying anesthesia nurses in two hospitals in Sweden found that parental involvement helps reduce anxiety, supports effective pain management, facilitates adaptation to the postoperative environment, and contributes to well-informed discharge decisions [20].

A systematic review and meta-analysis on more than 600 pediatric patients further demonstrated that the presence of parents in the post-anesthesia care unit can decrease the incidence of emergence delirium [21].

##### Additional Influencing Factors

Preoperative anxiety in young children undergoing surgery has been associated with increased postoperative pain, a higher incidence of anxiety, emergence delirium, sleep disturbances, and other problems [22]. A randomized controlled trial by Kain et al. in 2007, involving 408 children, demonstrated that the overall recovery experience can be improved through preoperative interventions, including comprehensive surgical preparation, child-friendly clinical practices, anxiety reduction strategies, parental presence during anesthetic induction, and the administration of premedication (e.g., dexmedetomidine) [23].

### 3.2. Airway and Respiration

Pediatric patients are uniquely vulnerable to respiratory complications in the postoperative period due to a combination of metabolic and anatomical factors [1]. Infants have a metabolic oxygen demand that is approximately twice that of adults. When faced with sustained hypoxia, they are at an increased risk of hypoxic respiratory depression. Anatomical characteristics, such as a softer and longer epiglottis, a compliant thoracic cage limiting the effective use of accessory respiratory muscles, immature protective reflexes, and a smaller functional residual capacity contribute to rapid desaturation when muscle tone is reduced.

The risk of apnea and bradycardia following surgical procedures is particularly pronounced in neonates born before 36 weeks of gestational age and remains elevated until a post-conceptual age of 56–60 weeks [24]. Monitoring respiratory effort and airway function in these high-risk patients should include clinical parameters such as breathing patterns, respiratory rate, and breath sounds, as well as technical measures like continuous pulse oximetry and electrocardiography, ideally including overnight monitoring.

Prophylactic and therapeutic administration of caffeine at doses of 5–10 mg/kg body weight has been shown to reduce the frequency of apnea in a prospective double-blind randomized trial in former premature infants [25]. Caffeine exerts its effects by stimulating the respiratory and cardiovascular systems, increasing responsiveness to arterial carbon dioxide tension, and enhancing diaphragmatic contractility. However, its use is associated with increased oxygen consumption and metabolic rate [26,27].

In early childhood, the enlargement of tonsils and adenoids may predispose children to airway obstruction and obstructive sleep apnea syndrome (OSAS). In non-airway surgery, this necessitates a multimodal approach to pain management, which may include perioperative regional anesthesia and cautious opioid titration, as pediatric patients with OSAS exhibit an increased respiratory sensitivity to opioids [28]. Notably, a recent systematic review found no additional benefit of local or regional anesthesia for pain management in tonsillotomy procedures [29].

A large prospective study on perioperative morbidity in children by Murat et al. in 2004 suggested that children undergoing airway surgeries, such as adenotonsillectomy or tonsillotomy, require vigilant postoperative monitoring, particularly if opioid analgesia is employed [1].

According to a review of high-risk ambulatory pediatric patients from 2023, scheduling surgeries for pediatric OSAS patients early in the day is advantageous, allowing for consistent and extended monitoring in the postoperative recovery unit [30].

The use of a laryngeal mask airway (LMA) has been shown to support airway patency during recovery. As early as 1997, Parry et al. recommend leaving the LMA in place until it is spontaneously expelled by the child, which reduces the need for additional airway maneuvers [31]. This practice has been successfully implemented in numerous hospitals. Spontaneously breathing children should initially be positioned in the lateral decubitus position until fully conscious, as it appears to be associated with less airway obstruction and fewer respiratory adverse events at the time of airway device removal in children [32]. Once stabilized, younger children should transition to the supine position, provided this positioning does not conflict with surgical or anesthetic considerations.

To predict the likelihood of postoperative respiratory complications in infants and children, a validated score, such as the Score for Prediction of Postoperative Respiratory Complications in Pediatric Patients (SPORC) [33], can be used. The eleven predictors, age < 2 yr, ASA physical status > 3, obesity or underweight, thoracic surgery, high dose of neuromuscular blocking agent, expected case duration ≥ 2 h, emergency surgery, upper airway infection within 7 days before surgery, congenital anomalies of nervous or cardiovascular system, congenital anomalies of the respiratory system, and being born as a preterm or small for gestational age infant have been shown to be predictive of an increased incidence of complications such as respiratory infection, aspiration pneumonitis, bronchospasm, laryngospasm, or oxygen desaturation.

Monitoring strategies and the duration of recovery room observation should be tailored accordingly.

#### Postoperative Residual Neuromuscular Blockade

Postoperative residual neuromuscular blockade remains a significant concern in the recovery room. A recent prospective audit of pediatric patients in 2015 reported an incidence of 28.1%, and that of a severe residual blockade, defined as a TOF ratio < 0.7, of 6.5% [34]. This condition warrants careful postoperative attention, as it may further decrease functional residual capacity and compromise protective pharyngeal and respiratory reflexes, which are essential for maintaining airway patency and respiratory drive [35].

Note: After reversal of steroidal muscle relaxants with sugammadex, children under the age of 2 years are predominantly at risk of experiencing postoperative residual or recurrent weakness [36].

### 3.3. Hypothermia

Hypothermia in the recovery room poses significant challenges to outcomes in pediatric populations. Heier et al. showed in 2006 that a reduction of 2 °C in body temperature can substantially extend the effects of muscle relaxants by doubling the duration of neuromuscular blockade [37]. Moreover, a decrease of 1 °C elevates postoperative oxygen consumption fourfold, exacerbates acidosis, and prolongs the effects of various drugs due to diminished kidney function and metabolism, which decline by approximately 7% for each 1 °C drop in temperature [38,39].

In term infants, hypothermia induces increases in heart rate, blood pressure, and catecholamine levels, whereas in preterm infants, it may result in bradycardia and reduced cardiac output [40].

A recent retrospective study of 1091 neonates and infants found that these physiological changes contributed to prolonged recovery room stays, increased admissions to intensive care units (ICUs), and extended overall hospitalization durations [41].

Notably, thermogenic shivering—a critical mechanism for generating heat—occurs only in children older than 6 years of age [42].

Note: Postoperative hypothermia can lead to the life-threatening triad of hypoxemia, metabolic acidosis, and hypoglycemia in neonates [43]. It is associated with delayed awakening, impaired protective reflexes, increased frequency of apneas, wound infections, coagulation disorders, elevated blood loss, intraventricular cerebral hemorrhage, arrhythmia, prolonged hospitalization, and poorer neurological outcomes [44].

### 3.4. Postoperative Nausea and/or Vomiting (PO(N)V)

PO(N)V is a common complication in pediatric patients, with an untreated incidence reaching up to 80% [45]. The ability to explicitly report nausea is typically possible only from around 4–5 years of age; thus, in younger children, the assessment predominantly focuses on postoperative vomiting (POV). Its occurrence can lead to dehydration, electrolyte disturbances, increased pain, and compromised surgical outcomes [46].

**Figure 1:**
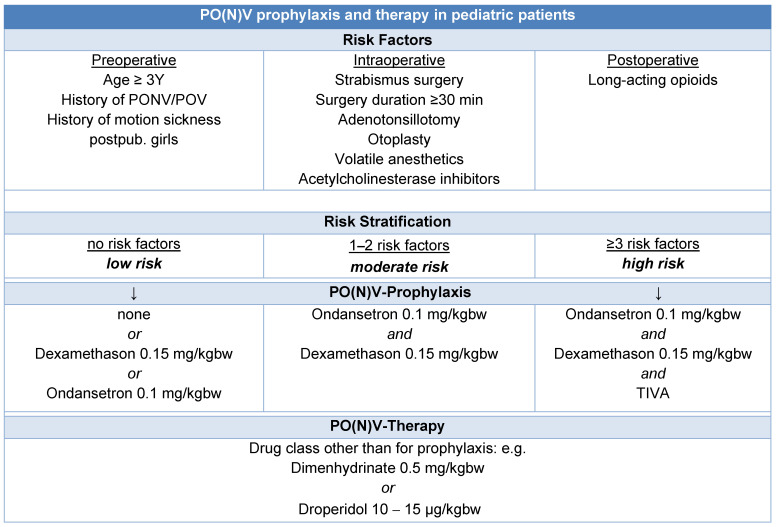
Algorithm for PO(N)V prophylaxis and therapy in pediatric patients [47,48].

Preventive strategies for PO(N)V include avoiding volatile anesthetics and nitrous oxide, utilizing total intravenous anesthesia with propofol, minimizing opioid use through regional anesthesia techniques, and ensuring adequate intraoperative hydration. Current guidelines recommend dual prophylaxis for PO(N)V in children over three years old or those presenting with one or two risk factors [47] (Figure 1).

While the effectiveness of alternative therapies such as acupuncture, acupressure, and transcutaneous nerve stimulation for managing PO(N)V in pediatric patients remains inadequately supported by evidence [49,50], a controlled randomized trial has shown that early postoperative intake of clear fluids [51] or ice popsicles can reduce the incidence of PO(N)V and improve overall well-being [52].

### 3.5. Emergence Delirium (ED)

With an incidence of up to 80%, ED poses a significant challenge in the recovery room, particularly in preschool-aged children and those undergoing head and neck surgeries—both non-modifiable risk factors [53]. ED is characterized by acute disturbances in consciousness and attention, distinct from postoperative agitation [54]. Accurate identification is crucial, as affected children may unintentionally engage in irrational behaviors that increase the risk of self-injury, necessitate additional nursing care, or delay hospital discharge [55].

**Figure 2:**
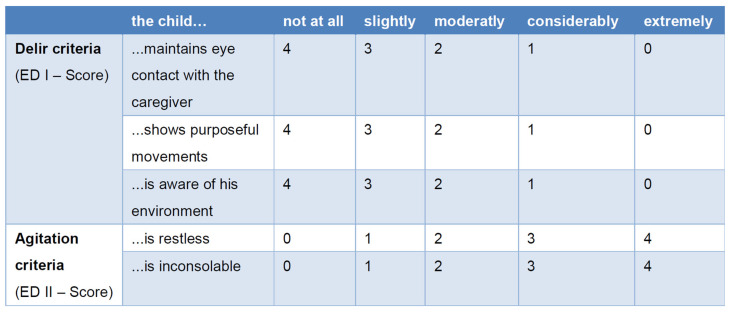
Pediatric Anesthesia Emergence Delirium (PAED) scale [53,57,60,61].

The Pediatric Anesthesia Emergence Delirium (PAED) scale is a validated diagnostic tool for quantifying the severity of ED in children [56]. It consists of five items [57] and can be divided into a delirium-specific score (ED I) and a nonspecific score (ED II) [58,59] (Figure 2).

Currently, a cutoff of ≥6 has been recommended to ensure that no children with ED are missed, despite the increased risk of false positive cases [61]. In contrast, an ED I score ≥ 9 has demonstrated a sensitivity of 0.93 and a specificity of 0.94 [60].

Effective strategies for reducing the incidence of ED include minimizing preoperative anxiety [62] through thorough family preparation and engaging children with digital media or music, both before [22,63] and during surgery [64].

Anesthesia management plays a pivotal role in prevention. Total intravenous anesthesia, particularly with a propofol bolus or a brief infusion of up to 3 mg/kg shortly before discharge to the recovery room, has shown efficacy in a meta-analysis of RCTs [65]. Additionally, a calm recovery environment, effective analgesia [66,67,68], and early intake of clear fluids contribute significantly to the child’s well-being and the reduction in ED episodes.

According to Davies et al., initial evidence suggests that intraoperative electroencephalographic (EEG) monitoring can enhance the prediction and prevention of ED. Anesthesiologists may reduce ED by timing patient emergence from anesthesia to coincide with appropriate EEG patterns indicative of natural sleep transitions [69].

Pharmacological interventions with propofol, 0.5–1 mg/kgbw, or, alternatively, ketamine S, 1 mg/kgbw iv, have demonstrated clinical success. Clonidine or dexmedetomidine can be administered in a dosage of 1–2 µg/kgbw for both prophylaxis and therapy [70].

A systematic review and meta-analysis of randomized controlled trials in 2022 found that administering magnesium sulfate also reduced the occurrence and severity of emergence agitation or delirium in children following general anesthesia [71].

### 3.6. Pain Assessment and Pain Management

Recent results from a study of registry data from 11 European hospitals continue to highlight substantial deficits in the concept, application, and dosing of analgesics in pediatric patients following surgery [72].

**Figure 3:**
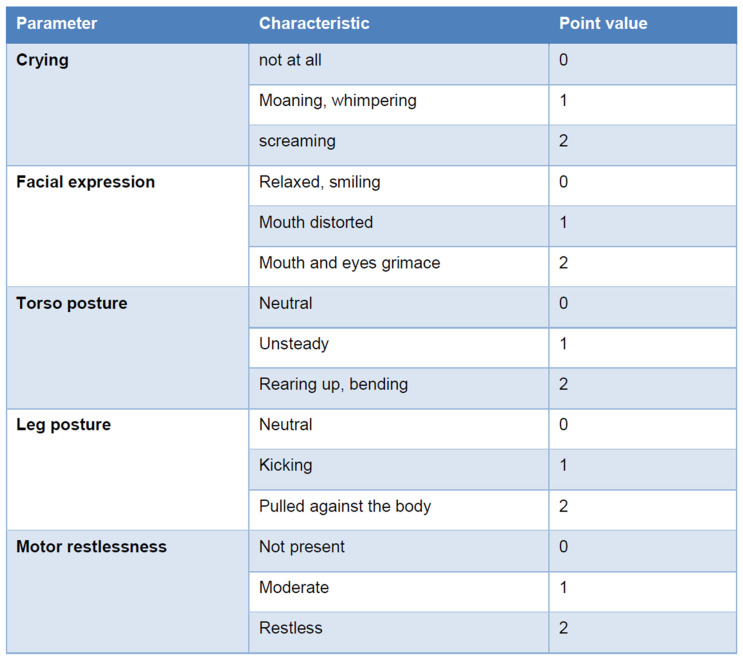
Children’s and Infants’ Postoperative Pain Scale (CHIPPS). The score value is calculated by adding the individual parameter point values after observing the child for 15 s. The need for analgesic therapy starts at four points [73].

Effective pain management in the recovery room requires the timely administration of adequate analgesic doses, and the use of standardized pain assessment scales tailored to all age groups. For children under four years of age, external assessment tools are essential, as verbalization of discomfort is often ineffective in this age group. Reliable and validated tools such as the revised Face, Legs, Activity, Cry, and Consolability (r-FLACC) scale and the Children’s and Infants’ Postoperative Pain Scale (CHIPPS) are particularly suited for this purpose [73,74,75,76] (Figure 3).

#### 3.6.1. Prevention of Chronic Post-Surgical Pain (CPSP)

According to Furuta et al., CPSP presents a significant challenge in pediatric care, occurring in 6–30% of cases within 3 to 12 months postoperatively [77]. CPSP is associated with considerable pain-related disabilities and increased healthcare costs. Preventative strategies emphasize comprehensive perioperative and postoperative pain management during the recovery phase, including techniques such as regional anesthesia.

Preoperative factors, including preexisting pain, anxiety, depressive symptoms, and sleep disturbances [77,78], have been considered potential risk factors for CPSP.

A recent systematic review and meta-analysis identified pre-surgical pain as the only preoperative risk factor that significantly predicted pediatric CPSP, emphasizing the critical need for effective pain management throughout the perioperative period, beginning before surgery [79].

#### 3.6.2. Non-Opioid Analgesics (NOPAs)

**Figure 4:**
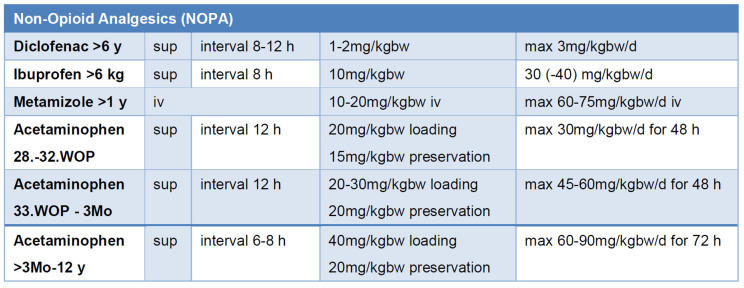
Dosing of non-opioid analgesics (NOPAs) to achieve effective drug levels [82,83]; bw: body weight, iv: intravenous, sup: suppositorium, WOP: week of pregnancy.

Nonsteroidal anti-inflammatory drugs (NSAIDs) are a cornerstone of pediatric pain management and are commonly used in infants, children, and adolescents [80]. In many RCTs, they demonstrate superior efficacy compared to acetaminophen and, when combined with opioids, reduce required opioid dosages while enhancing analgesic efficacy and minimizing adverse effects such as vomiting and sedation [81,82] (Figure 4).

As NSAIDs impair platelet and kidney function in children, they can lead to ductus arteriosus occlusion and increase the risk of necrotizing enterocolitis or intraventricular hemorrhage [84,85].

Acetaminophen is widely used, including in neonates, though caution is required due to potential hepatotoxicity. To maximize analgesic potential and ensure safe dosing, age, body weight, therapy duration, maximum daily dose, and dosing intervals must be considered [82,86,87] (Figure 4).

Metamizole, despite its controversial status, offers minimal respiratory depression and is effective in managing visceral pain [88,89].

Consistent documentation of medication timing and dosage is particularly critical during the transfer to a peripheral ward to prevent both overdosing and prolonged treatment intervals.

#### 3.6.3. Opioids

Piritramide and morphine are effective postoperative analgesics, with piritramide preferred due to its stable hemodynamic profile and lower incidence of pruritus. Patient-controlled analgesia (PCA) by proxy remains a safe and efficient method of pain administration for pediatric patients, provided that limitations like developmental and neurological disabilities are respected [90].

Opioids in newborns and premature infants pose unique challenges as they act more strongly in the brainstem than in the midbrain, where the modulation of emotional pain perception and cortical pain transmission is mediated. Therefore, they lead to significant respiratory depression at levels that are insufficient for analgesia.

Note: Newborns and premature babies can exhibit pronounced respiratory depression at opioid levels that do not provide sufficient analgesia. Opioid titration to analgesia without respiratory depression is, therefore, not possible [91].

Nalbuphine, a functional µ-antagonist and κ-agonist, is suitable for the management of mild to moderate pain. It has a pronounced sedative effect without causing respiratory depression. At a dosage of 0.3–0.4 mg/kgbw iv, nalbuphine demonstrates a ceiling effect, preventing further analgesic enhancement [92].

For tonsillectomy, systematic reviews have identified the combination of acetaminophen, NSAIDs, and dexamethasone as the most effective approach, whereas regional or local anesthesia provides no additional benefit in this context [29,93].

#### 3.6.4. Co-Analgesics

Dexamethasone, clonidine, and dexmedetomidine are well-established co-analgesics that reduce opioid requirements while effectively managing PO(N)V and pain [94].

Gabapentinoids such as gabapentin and pregabalin have shown promise in reducing pain, nausea, and emergence delirium in specific pediatric populations (e.g., after adenotonsillectomy or posterior spinal fusion). While side effects like sedation and respiratory depression are common in adults, they appear to be less frequent in children [95].

Non-pharmacological interventions can further alleviate pain and reduce reliance on analgesics, thereby minimizing their adverse effects. Techniques such as tablet use, mobile phones, pacifiers, and caregiver presence are beneficial across various pediatric age groups. In preterm neonates, interventions like non-nutritive sucking, facilitated tucking, and swaddling help reduce pain behaviors. In full-term neonates, non-nutritive sucking is particularly effective. However, evidence indicates that none of these interventions is promising in reducing pain in older infants [96].

For inpatient settings, the ESPA Pain Management Ladder Initiative offers guidance to encourage optimal pain management practice and to support institutions in developing individual pain management concepts according to their financial and human resources [97,98].

### 3.7. Early Postoperative Clear Fluid Intake

When clinically appropriate, permitting early oral intake after surgery has been shown to enhance patient satisfaction, reduce opioid consumption, and decrease the incidence of nausea and vomiting [51]. The administration of small portions of water ice, typically around 40 mL, is particularly effective and well tolerated, even in very young children, with virtually no contraindications.

For school-aged children and older, chewing gum can serve as a valuable adjunct by providing distraction, improving well-being, and alleviating nausea. However, a systematic review and meta-analysis demonstrated that gum chewing is not associated with earlier postoperative gastrointestinal recovery in children [99].

Note: A multimodal analgesia approach—integrating regional anesthesia, non-opioid analgesics, opioids, and co-analgesics—remains a cornerstone to effective postoperative anesthetic care [100].

### 3.8. Discharge Criteria from Postoperative Recovery Room Care

Readiness for discharge from postoperative care should be evaluated using standardized criteria and formally documented.

For inpatients, discharge readiness requires an adequate level of consciousness, sufficient pain control, stable vital signs, and vital functions within the capabilities of the receiving ward.

For patients being discharged from the monitoring unit without further medical supervision, the criteria include the child being awake, oriented, exhibiting normal motor function, and having intact protective reflexes as initially observed. Stable, unassisted breathing with a peripheral oxygen saturation (SpO_2_) above 95% without supplemental oxygen for at least one hour, the absence of stridor, stable vital signs, and controlled pain (numerical rating scale [NRS] < 4) are prerequisites. Further prerequisites include the absence of PO(N)V, the ability to drink fluids, non-irritated and non-bleeding wound sites [101], and normothermia.

The revised Aldrete Score, which assesses activity, respiration, circulation, consciousness, and oxygen saturation, is a valuable tool for evaluating discharge readiness. However, it does not encompass pain or PO(N)V—both critical factors in evaluating pediatric recovery [101].

Continuous nerve blocks have demonstrated safety, efficacy, and high satisfaction rates in the treatment of acute pain among both patients and caregivers. This was found not only in perioperative and recovery unit settings, but also in outpatient care across various countries [102,103,104].

For infants under six months of age undergoing ambulatory procedures, strict adherence to a two-hour discharge criterion is not necessary, as it does not enhance safety, provided physiological readiness for discharge is achieved and patient selection is performed judiciously [105].

Note: The implementation of quality improvement measures—such as streamlined, structured handovers, standardized care protocols with checklists, and continuous healthcare provider training—remains integral to reducing translational gaps and enhancing safety and outcomes in postoperative pediatric recovery room care [4,106].

## 4. Conclusions

High-quality care in the pediatric recovery room is paramount, as it directly impacts perioperative safety, patient outcomes, overall hospital experience, and healthcare costs. Achieving this standard requires recovery units equipped with monitoring systems tailored to the specific needs of pediatric patients, and staffed by healthcare professionals with specialized training. Respect for the child’s privacy and the presence of parents are essential elements of patient-centered care.

Key strategies to optimize pediatric recovery include early oral fluid intake, effective and individualized pain management, and strategies to alleviate pain, anxiety, and stress. Additionally, the prevention and prompt treatment of postoperative nausea and vomiting and emergence delirium are critical for ensuring a smooth recovery.

Organizational measures, such as structured handovers, the implementation of standardized care protocols, ongoing professional development, quality improvement initiatives, and strategies for integrating new evidence, serve as foundational pillars for enhancing safety, accelerating recovery, and ensuring high levels of patient and caregiver satisfaction.

## Data Availability

Not applicable.

## Abbreviations

The following abbreviations are used in this manuscript:

CHIPPS	Children’s and Infants’ Postoperative Pain Scale
CPSP	chronic post-surgical pain
ECG	electrocardiographic
ED	emergence delirium
EEG	electroencephalographic
LMA	laryngeal mask airway
NOPA	non-opioid analgesic
NSAID	nonsteroidal anti-inflammatory drugs
OSAS	obstructive sleep apnea syndrome
PO(N)V	postoperative nausea and/or vomiting
r-FLACC scale	revised Face, Legs, Activity, Cry, and Consolability scale
